# Supplementary material for: The relationship between the severity and complications of Henöch-Schönlein purpura in children and dietary inflammatory index: a retrospective cohort study
Source: PeerJ. 2024 Sep 24;12:e18175. doi: 10.7717/peerj.18175 (PMC11430262; doi:10.7717/peerj.18175)
Supplement: Supplemental Information 2 [file peerj-12-18175-s002.docx]

|  | **0** | **1** | **2** | **3** |
| --- | --- | --- | --- | --- |
| Gender (Male/Female) | Male | Female | - | - |
| Allergic Purpura Family History (Y/N) | Negative | Positive | - | - |
| Mode of Delivery | Cesarean Section | Vaginal Delivery | - | - |
| Renal complications | Negative | Positive | - | - |
| Skin complications | Negative | Positive | Alcoholic | Hyperlipidemic |
| Gastrointestinal complications | Negative | Positive |  |  |
| Coagulation disorders | Negative | Positive |  |  |
| Respiratory complications | Negative | Positive |  |  |
| Neurological complications | Negative | Positive |  |  |
