# Supplementary material for: The relationship between the severity and complications of Henöch-Schönlein purpura in children and dietary inflammatory index: a retrospective cohort study
Source: PeerJ. 2024 Sep 24;12:e18175. doi: 10.7717/peerj.18175 (PMC11430262; doi:10.7717/peerj.18175)
Supplement: Supplemental Information 3 [file peerj-12-18175-s003.docx]

STROBE Statement—checklist of items that should be included in reports of observational studies

|  | Item No. | Recommendation | Page  No. | Relevant text from manuscript |
| --- | --- | --- | --- | --- |
| **Title and abstract** | 1 | (*a*) Indicate the study’s design with a commonly used term in the title or the abstract | 1 | A retrospective cohort study was conducted, enrolling children aged 2-14 years diagnosed with HSP. |
|  |  | (*b*) Provide in the abstract an informative and balanced summary of what was done and what was found | 1 | The study highlights the potential influence of dietary inflammatory potential, as quantified by the DII, on disease severity and complications in children with HSP. Understanding the interplay between dietary patterns and inflammatory responses in pediatric vasculitis has implications for the management of HSP, emphasizing the relevance of considering dietary interventions to optimize clinical outcomes and improve the overall well-being of affected children. |
| Introduction | | | |  |
| Background/rationale | 2 | Explain the scientific background and rationale for the investigation being reported | 2-3 | Henöch-Schönlein purpura (HSP) is a systemic vasculitis characterized by the deposition of immune complexes containing predominantly IgA in small vessels throughout the body, leading to inflammation and tissue damage(1-3). The classic tetrad of HSP encompasses palpable purpura (small, red to purple skin spots that are palpable), arthritis or arthralgia, gastrointestinal symptoms (such as abdominal pain, vomiting, and gastrointestinal bleeding), and nephritis (manifesting as hematuria and proteinuria)(4-6).  HSP primarily affects children, with the majority of cases reported in those between 2 and 6 years of age(7-9). While the exact etiology of HSP remains incompletely understood, it is believed to involve an aberrant immune response triggered by various environmental factors, infectious agents, and genetic predisposition(6, 10, 11). The clinical course of HSP is highly variable, ranging from a self-limiting and benign condition to severe and persistent disease with renal involvement and long-term complications(4, 12, 13).  The systemic nature of HSP, with its potential to affect multiple organs and systems, underscores the significance of understanding the factors that may modulate disease severity and outcomes(14). The association between dietary factors, systemic inflammation, and immune dysregulation has garnered increasing attention in the context of various inflammatory conditions, raising the question of whether dietary inflammatory potential may contribute to the clinical manifestations and progression of HSP(3).  In recent years, emerging evidence has suggested that dietary patterns and their inflammatory potential may play a significant role in modulating the immune response and disease outcomes in various inflammatory conditions, including vasculitis. The Dietary Inflammatory Index (DII) is a composite scoring algorithm developed to quantify the overall inflammatory potential of an individual's diet based on the intake of specific nutrients and bioactive compounds(15-17). A higher DII score reflects a more pro-inflammatory diet, while a lower score indicates a more anti-inflammatory diet(18-20). Several epidemiological studies have demonstrated the association between high DII and an increased risk of chronic inflammatory conditions, such as cardiovascular diseases, metabolic syndrome, and certain cancers(21-23). |
| Objectives | 3 | State specific objectives, including any prespecified hypotheses | 3 | Despite the growing understanding of the potential impact of diet on systemic inflammation and disease susceptibility, limited research has focused on the role of dietary inflammatory potential in pediatric vasculitis, particularly HSP. Therefore, this study aims to investigate the association between DII and disease severity as well as complications in children diagnosed with HSP. The findings may contribute to a deeper understanding of the interplay between dietary patterns and inflammatory responses in pediatric vasculitis, ultimately paving the way for novel dietary-based interventions in the management of HSP. |
| Methods | | | |  |
| Study design | 4 | Present key elements of study design early in the paper | 4 | This was a retrospective cohort study. The cohort included children 2 to 14 years of age who have received a diagnosis of HSP were recruited from the clinical data database of this hospital. The study period was from April 2021 to March 2024. The cohort was divided into two groups according to the dietary inflammation index score: low dietary inflammation index group and high dietary inflammation index group. |
| Setting | 5 | Describe the setting, locations, and relevant dates, including periods of recruitment, exposure, follow-up, and data collection | 4 | This was a retrospective cohort study. The cohort included children 2 to 14 years of age who have received a diagnosis of HSP were recruited from the clinical data database of this hospital. The study period was from April 2021 to March 2024. The cohort was divided into two groups according to the dietary inflammation index score: low dietary inflammation index group and high dietary inflammation index group. |
| Participants | 6 | (*a*) *Cohort study*—Give the eligibility criteria, and the sources and methods of selection of participants. Describe methods of follow-up  *Case-control study*—Give the eligibility criteria, and the sources and methods of case ascertainment and control selection. Give the rationale for the choice of cases and controls  *Cross-sectional study*—Give the eligibility criteria, and the sources and methods of selection of participants | 4 | Inclusion Criteria: Children aged 2-14 years diagnosed with HSP (3); Availability of complete dietary records for a period of at least six months prior to the diagnosis of HSP; Availability of follow-up data for at least six months post-diagnosis; Parents or legal guardians were willing to provide informed consent for their children to participate in this study.This study was approved by the Ethics Committee of Hainan Women and Children's Medical Center and complies with the ethical guidelines of the Helsinki Declaration (No.HNWCMC-2021-38).  Exclusion Criteria: Children with co-existing significant chronic inflammatory conditions, such as inflammatory bowel disease, juvenile rheumatoid arthritis, or systemic lupus erythematosus, which may confound the relationship between dietary factors and disease severity in HSP; Children with a history of major dietary changes or modifications during the study period, which may substantially affect the accuracy and reliability of the dietary records. |
|  |  | (*b*) *Cohort study*—For matched studies, give matching criteria and number of exposed and unexposed  *Case-control study*—For matched studies, give matching criteria and the number of controls per case |  |  |
| Variables | 7 | Clearly define all outcomes, exposures, predictors, potential confounders, and effect modifiers. Give diagnostic criteria, if applicable | 5 | Data on dietary intake will be collected through validated food frequency questionnaires and dietary records. Information on disease severity and complications will be obtained from medical records, including laboratory results, clinical assessments, and diagnostic imaging reports. Other relevant demographic and clinical data will also be collected, including age, sex, comorbidities, and medications. |
| Data sources/ measurement | 8* | For each variable of interest, give sources of data and details of methods of assessment (measurement). Describe comparability of assessment methods if there is more than one group | *5* | *Data on dietary intake will be collected through validated food frequency questionnaires and dietary records. Information on disease severity and complications will be obtained from medical records, including laboratory results, clinical assessments, and diagnostic imaging reports. Other relevant demographic and clinical data will also be collected, including age, sex, comorbidities, and medications.* |
| Bias | 9 | Describe any efforts to address potential sources of bias | 6 | The demographic and clinical characteristics of the cohort were analyzed using SPSS 25.0 statistical software. Descriptive statistics were presented for categorical data as [n(%)] when the sample size was ≥40 and the theoretical frequency (T) was ≥5. The chi-square test with the basic formula was used as the test statistic (χ2) for these cases. However, if the sample size was ≥40 but the theoretical frequency was 1≤T<5, the chi-square test was adjusted using the correction formula. When the sample size was <40 or the theoretical frequency (T) was <1, statistical analysis was performed using Fisher's exact test. For normally distributed continuous data, the mean and standard deviation were presented as (x±s). For non-normally distributed data, statistical analysis was carried out after variable transformation to achieve normal distribution, and the t-test was used. The relationship between DII and disease severity as well as complications was assessed using Spearman's correlation analysis, and statistically significant differences between the two groups were identified. A significance level of P＜0.05 was used to indicate statistical significance. |
| Study size | 10 | Explain how the study size was arrived at | 7 | A total of 115 subjects were divided into Anti-inflammatory dietary group (n=56) and Pro-inflammatory dietary group (n=59). |

Continued on next page

| Quantitative variables | 11 | Explain how quantitative variables were handled in the analyses. If applicable, describe which groupings were chosen and why | 6 | The demographic and clinical characteristics of the cohort were analyzed using SPSS 25.0 statistical software. Descriptive statistics were presented for categorical data as [n(%)] when the sample size was ≥40 and the theoretical frequency (T) was ≥5. The chi-square test with the basic formula was used as the test statistic (χ2) for these cases. However, if the sample size was ≥40 but the theoretical frequency was 1≤T<5, the chi-square test was adjusted using the correction formula. When the sample size was <40 or the theoretical frequency (T) was <1, statistical analysis was performed using Fisher's exact test. For normally distributed continuous data, the mean and standard deviation were presented as (x±s). For non-normally distributed data, statistical analysis was carried out after variable transformation to achieve normal distribution, and the t-test was used. The relationship between DII and disease severity as well as complications was assessed using Spearman's correlation analysis, and statistically significant differences between the two groups were identified. A significance level of P＜0.05 was used to indicate statistical significance. |
| --- | --- | --- | --- | --- |
| Statistical methods | 12 | (*a*) Describe all statistical methods, including those used to control for confounding | 6 | The demographic and clinical characteristics of the cohort were analyzed using SPSS 25.0 statistical software. Descriptive statistics were presented for categorical data as [n(%)] when the sample size was ≥40 and the theoretical frequency (T) was ≥5. The chi-square test with the basic formula was used as the test statistic (χ2) for these cases. However, if the sample size was ≥40 but the theoretical frequency was 1≤T<5, the chi-square test was adjusted using the correction formula. When the sample size was <40 or the theoretical frequency (T) was <1, statistical analysis was performed using Fisher's exact test. For normally distributed continuous data, the mean and standard deviation were presented as (x±s). For non-normally distributed data, statistical analysis was carried out after variable transformation to achieve normal distribution, and the t-test was used. The relationship between DII and disease severity as well as complications was assessed using Spearman's correlation analysis, and statistically significant differences between the two groups were identified. A significance level of P＜0.05 was used to indicate statistical significance. |
|  |  | (*b*) Describe any methods used to examine subgroups and interactions | NA | NA |
|  |  | (*c*) Explain how missing data were addressed | NA | NA |
|  |  | (*d*) *Cohort study*—If applicable, explain how loss to follow-up was addressed  *Case-control study*—If applicable, explain how matching of cases and controls was addressed  *Cross-sectional study*—If applicable, describe analytical methods taking account of sampling strategy | 6 | The demographic and clinical characteristics of the cohort were analyzed using SPSS 25.0 statistical software. Descriptive statistics were presented for categorical data as [n(%)] when the sample size was ≥40 and the theoretical frequency (T) was ≥5. The chi-square test with the basic formula was used as the test statistic (χ2) for these cases. However, if the sample size was ≥40 but the theoretical frequency was 1≤T<5, the chi-square test was adjusted using the correction formula. When the sample size was <40 or the theoretical frequency (T) was <1, statistical analysis was performed using Fisher's exact test. For normally distributed continuous data, the mean and standard deviation were presented as (x±s). For non-normally distributed data, statistical analysis was carried out after variable transformation to achieve normal distribution, and the t-test was used. The relationship between DII and disease severity as well as complications was assessed using Spearman's correlation analysis, and statistically significant differences between the two groups were identified. A significance level of P＜0.05 was used to indicate statistical significance. |
|  |  | (*e*) Describe any sensitivity analyses | NA | NA |
| Results | | | | |
| Participants | 13* | (a) Report numbers of individuals at each stage of study—eg numbers potentially eligible, examined for eligibility, confirmed eligible, included in the study, completing follow-up, and analysed | 7 | In this study, we aimed to investigate the association between DII and demographic as well as clinical characteristics in the study population (Table 1). A total of 115 subjects were divided into Anti-inflammatory dietary group (n=56) and Pro-inflammatory dietary group (n=59). The analysis of demographic characteristics indicated comparable distribution with respect to age (8.62 ± 1.52 vs. 8.89 ± 1.75, p=0.387), body mass index (17.98 ± 2.05 vs. 18.16 ± 2.22, p=0.65), gender, disease duration (15.41 ± 6.83 vs. 16.75 ± 7.24, p=0.309), HSP family history, physical activity (3.52 ± 1.04 vs. 3.21 ± 0.88, p=0.09), and mode of delivery. The distribution of HSP family history (32.14% vs. 35.59%, p=0.846) as well as physical activity (1.71, p=0.09) did not show statistically significant differences between the two groups. Furthermore, no significant association was found between DII status and mode of delivery (p=1). These findings suggest that there were no significant differences in demographic and clinical characteristics between the low DII and Pro-inflammatory dietary groups, indicating a well-balanced study population. |
|  |  | (b) Give reasons for non-participation at each stage | NA | NA |
|  |  | (c) Consider use of a flow diagram | NA | NA |
| Descriptive data | 14* | (a) Give characteristics of study participants (eg demographic, clinical, social) and information on exposures and potential confounders | 7 | In this study, we aimed to investigate the association between DII and demographic as well as clinical characteristics in the study population (Table 1). A total of 115 subjects were divided into Anti-inflammatory dietary group (n=56) and Pro-inflammatory dietary group (n=59). The analysis of demographic characteristics indicated comparable distribution with respect to age (8.62 ± 1.52 vs. 8.89 ± 1.75, p=0.387), body mass index (17.98 ± 2.05 vs. 18.16 ± 2.22, p=0.65), gender, disease duration (15.41 ± 6.83 vs. 16.75 ± 7.24, p=0.309), HSP family history, physical activity (3.52 ± 1.04 vs. 3.21 ± 0.88, p=0.09), and mode of delivery. The distribution of HSP family history (32.14% vs. 35.59%, p=0.846) as well as physical activity (1.71, p=0.09) did not show statistically significant differences between the two groups. Furthermore, no significant association was found between DII status and mode of delivery (p=1). These findings suggest that there were no significant differences in demographic and clinical characteristics between the low DII and Pro-inflammatory dietary groups, indicating a well-balanced study population. |
|  |  | (b) Indicate number of participants with missing data for each variable of interest | NA | NA |
|  |  | (c) *Cohort study*—Summarise follow-up time (eg, average and total amount) | NA | NA |
| Outcome data | 15* | *Cohort study*—Report numbers of outcome events or summary measures over time |  |  |
|  |  | *Case-control study—*Report numbers in each exposure category, or summary measures of exposure | *7* | *In this study, we aimed to investigate the association between DII and demographic as well as clinical characteristics in the study population (Table 1). A total of 115 subjects were divided into Anti-inflammatory dietary group (n=56) and Pro-inflammatory dietary group (n=59). The analysis of demographic characteristics indicated comparable distribution with respect to age (8.62 ± 1.52 vs. 8.89 ± 1.75, p=0.387), body mass index (17.98 ± 2.05 vs. 18.16 ± 2.22, p=0.65), gender, disease duration (15.41 ± 6.83 vs. 16.75 ± 7.24, p=0.309), HSP family history, physical activity (3.52 ± 1.04 vs. 3.21 ± 0.88, p=0.09), and mode of delivery. The distribution of HSP family history (32.14% vs. 35.59%, p=0.846) as well as physical activity (1.71, p=0.09) did not show statistically significant differences between the two groups. Furthermore, no significant association was found between DII status and mode of delivery (p=1). These findings suggest that there were no significant differences in demographic and clinical characteristics between the low DII and Pro-inflammatory dietary groups, indicating a well-balanced study population.* |
|  |  | *Cross-sectional study—*Report numbers of outcome events or summary measures | *NA* | *NA* |
| Main results | 16 | (*a*) Give unadjusted estimates and, if applicable, confounder-adjusted estimates and their precision (eg, 95% confidence interval). Make clear which confounders were adjusted for and why they were included | NA | NA |
|  |  | (*b*) Report category boundaries when continuous variables were categorized | NA | NA |
|  |  | (*c*) If relevant, consider translating estimates of relative risk into absolute risk for a meaningful time period | NA | NA |

Continued on next page

| Other analyses | 17 | Report other analyses done—eg analyses of subgroups and interactions, and sensitivity analyses | 9-10 | Finally, the correlation analysis of the DII with disease severity and complications in children with HSP revealed significant positive correlations (Table 5). The DII demonstrated a moderate to strong positive correlation with various disease complications, including renal (r=0.21, p=0.024), skin (r=0.243, p=0.009), gastrointestinal (r=0.276, p=0.003), coagulation disorders (r=0.286, p=0.002), and respiratory complications (r=0.241, p=0.01). Moreover, DII exhibited a significant positive correlation with disease severity (r=0.284, p=0.002). Although the correlation with neurological complications was not statistically significant (r=0.171, p=0.068), the overall findings indicate a notable association between higher DII and increased disease severity as well as a higher risk of various complications in children with HSP. These results underscore the potential influence of dietary inflammatory potential on disease outcomes in pediatric patients. |
| --- | --- | --- | --- | --- |
| Discussion | | | | |
| Key results | 18 | Summarise key results with reference to study objectives | 10 | The findings of this study shed light on the potential influence of dietary factors, specifically the DII, on disease severity and complications in children diagnosed with HSP. The results revealed significant associations between higher DII and elevated inflammatory biomarkers, adverse nutrient intake, blood lipid profiles, disease complications, and disease severity. These findings provide valuable insights into the interplay between dietary patterns and inflammatory responses in pediatric vasculitis, specifically HSP, and have implications for the management and potential dietary-based interventions in HSP. |
| Limitations | 19 | Discuss limitations of the study, taking into account sources of potential bias or imprecision. Discuss both direction and magnitude of any potential bias | 11-12 | While the study provides important insights into the association between DII and disease severity and complications in children with HSP, several limitations should be acknowledged. The retrospective nature of the cohort study may introduce inherent biases, and the generalizability of the findings to broader populations should be approached with caution. Additionally, the study's reliance on self-reported dietary data and the use of a single dietary assessment tool may present limitations in capturing the comprehensive dietary habits of the participants. Future research could benefit from longitudinal prospective studies with larger sample sizes and more comprehensive dietary assessments to further elucidate the role of dietary inflammatory potential in pediatric vasculitis. |
| Interpretation | 20 | Give a cautious overall interpretation of results considering objectives, limitations, multiplicity of analyses, results from similar studies, and other relevant evidence | 11 | In conclusion, the findings of this study underscore the potential influence of dietary inflammatory potential, as quantified by the DII, on disease severity and complications in children diagnosed with HSP. The observed associations between higher DII and elevated inflammatory biomarkers, adverse nutrient intake, adverse lipid profiles, disease complications, and disease severity highlight the relevance of considering dietary patterns in the management of pediatric vasculitis. Moving forward, integrating dietary assessments and interventions as part of a comprehensive approach to managing pediatric vasculitis, including HSP, has the potential to optimize clinical outcomes and improve the overall well-being of affected children. Further research and multidisciplinary collaborations are warranted to explore the role of dietary interventions in mitigating disease severity and reducing the risk of complications in pediatric patients with vasculitis. |
| Generalisability | 21 | Discuss the generalisability (external validity) of the study results | 10 | The observed correlations between DII and disease severity, as well as various complications, have important clinical implications for the management of HSP in pediatric patients. Understanding the potential impact of dietary patterns on disease outcomes can provide healthcare professionals with valuable insights for developing tailored dietary interventions to complement traditional treatment approaches. Incorporating dietary assessments and interventions aimed at modulating the inflammatory potential of the diet could be beneficial in mitigating disease severity and reducing the risk of complications in children with HSP. |
| Other information | |  | | |
| Funding | 22 | Give the source of funding and the role of the funders for the present study and, if applicable, for the original study on which the present article is based | 12 | This study was supported by the Excellent Talent Team of Hainan Province (No.QRCBT202121); Hainan Province Clinical Medical Center (No.QWYH202175) and Natural Science Foundation of Hainan Province (No.821RC1130). |

*Give information separately for cases and controls in case-control studies and, if applicable, for exposed and unexposed groups in cohort and cross-sectional studies.

**Note:** An Explanation and Elaboration article discusses each checklist item and gives methodological background and published examples of transparent reporting. The STROBE checklist is best used in conjunction with this article (freely available on the Web sites of PLoS Medicine at http://www.plosmedicine.org/, Annals of Internal Medicine at http://www.annals.org/, and Epidemiology at http://www.epidem.com/). Information on the STROBE Initiative is available at www.strobe-statement.org.
